# Supplementary figures and images for: Induction of fetal hemoglobin: Lentiviral shRNA knockdown of HBS1L in β0-thalassemia/HbE erythroid cells
Source: PLoS One. 2023 Mar 8;18(3):e0281059. doi: 10.1371/journal.pone.0281059 (PMC9994754; doi:10.1371/journal.pone.0281059)

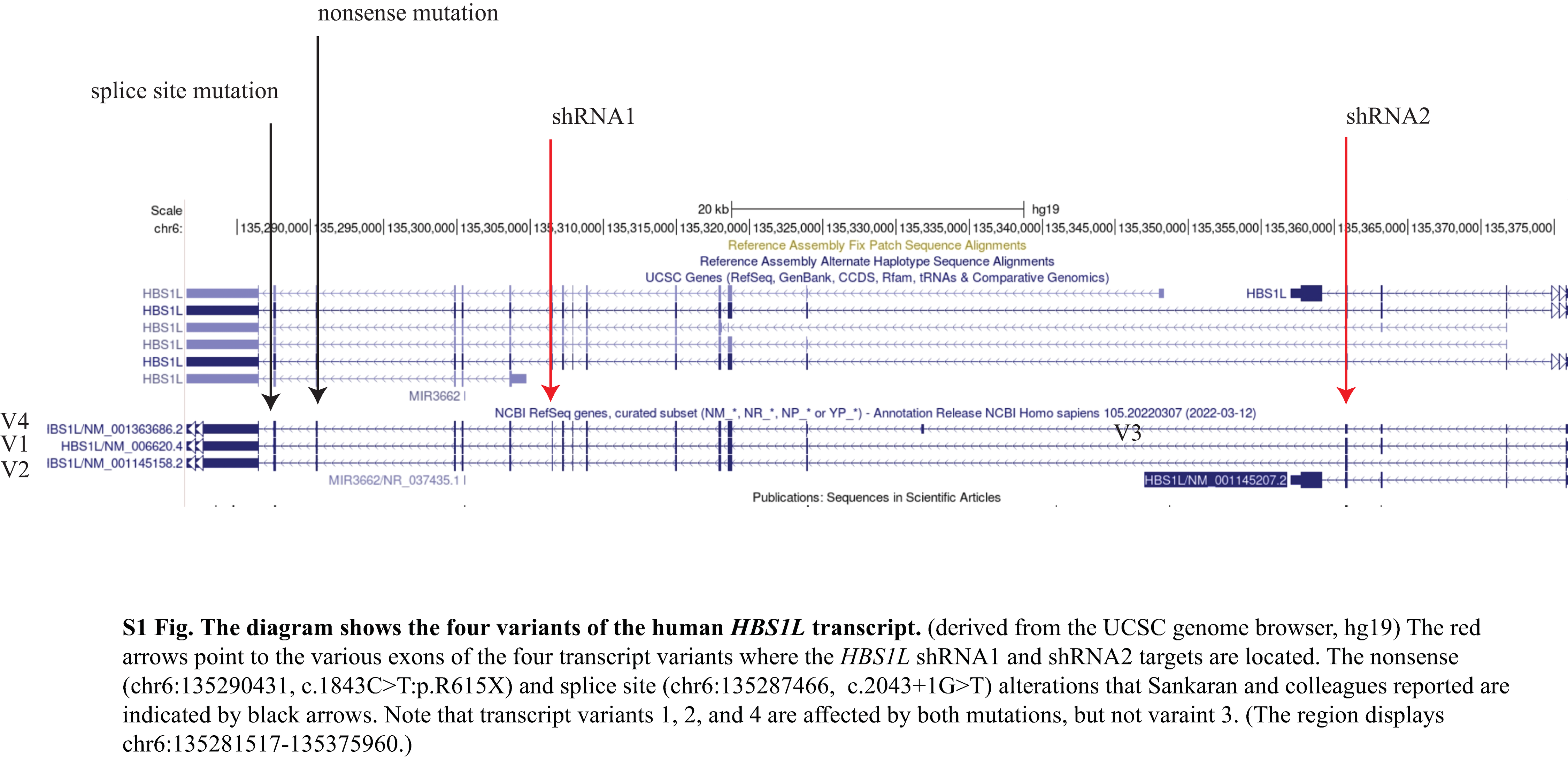

Supplement: S1 Fig — (TIF) [file pone.0281059.s001.tif]

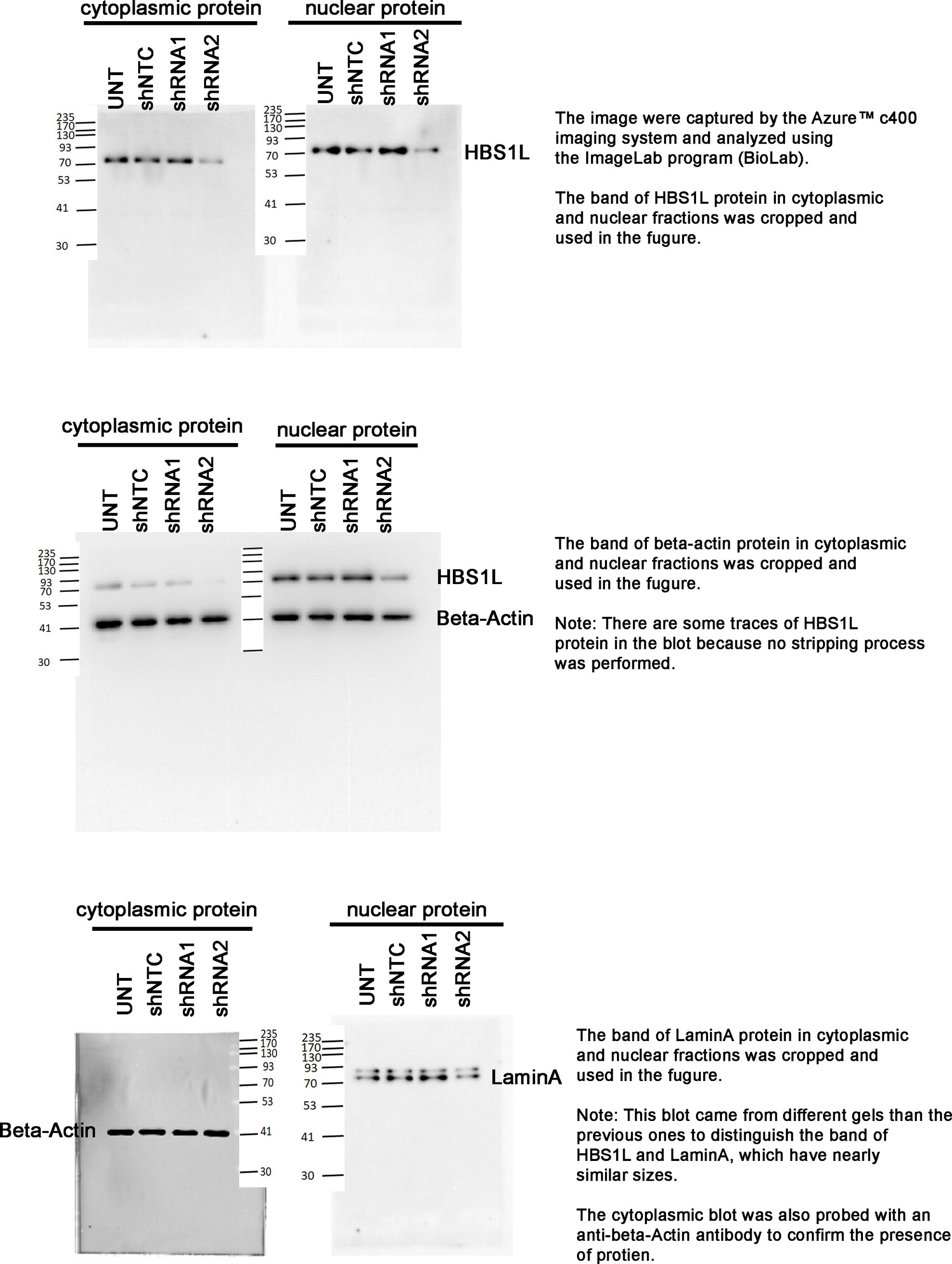

Supplement: S1 Raw images — (TIF) [file pone.0281059.s004.tif]
