# Supplementary material for: Induction of fetal hemoglobin: Lentiviral shRNA knockdown of HBS1L in β0-thalassemia/HbE erythroid cells
Source: PLoS One. 2023 Mar 8;18(3):e0281059. doi: 10.1371/journal.pone.0281059 (PMC9994754; doi:10.1371/journal.pone.0281059)
Supplement: S1 Table — (DOCX) [file pone.0281059.s002.docx]

**S1 Table 1 Clinical data of the healthy donors and β^0^-thalassemia/HbE subjects recruited in this study**

| **Code** | **Sex** | **Race** | **Age** | **β-globin gene** | **α-globin gene** |
| --- | --- | --- | --- | --- | --- |
| *HD1 | female | Thai | N/A | Normal | Normal |
| #*HD2 | female | Thai | 25 | Normal | Normal |
| *HD3 | female | Thai | 33 | Normal | Normal |
| #HD4 | female | Thai-Lao | 25 | Normal | Normal |
| #HD5 | female | Thai | 27 | Normal | Normal |
| *BE1 | female | Thai | 19 | Cod 17 (A>T) | Normal |
| #*BE2 | male | Lao Song | 30 | Cod 17 (A>T) | Normal |
| *BE3 | male | Thai | 32 | Cod 17 (A>T) | Normal |
| #BE4 | male | Thai | 44 | Cod 41/42 (-TTCT) | Normal |
| #BE5 | female | Thai-Chinese | 18 | Cod 41/42 (-TTCT) | Normal |

* The subjects were recruited in knockdown experiment.

# The subjects were recruited in expression study.

HD = healthy donor, BE = β^0^-thalassemia/HbE

N/A = not available
