# Supplementary material for: Induction of fetal hemoglobin: Lentiviral shRNA knockdown of HBS1L in β0-thalassemia/HbE erythroid cells
Source: PLoS One. 2023 Mar 8;18(3):e0281059. doi: 10.1371/journal.pone.0281059 (PMC9994754; doi:10.1371/journal.pone.0281059)
Supplement: S2 Table — (DOCX) [file pone.0281059.s003.docx]

**Supplementary Table 2.** List of primer sequences used for qPCR in this study.

| **Gene** | **Forward primer (5' to 3')** | **Reverse primer (5' to 3')** |
| --- | --- | --- |
| *ACTB* | GGC ACC ACA CCT TCT ACA ATG | GGT CTC AAA CAT GAT CTG GGT C |
| *HBS1L* | TAT CAG TAT CTA AGC GGA GTG | CAT CAT CTT CAA AAT CTT CAT CG |
| *BCL11A* | GGG AAT TCT CGC CCG AG | GGG AAG TTC ATC TGG CAC T |
| *ZBTB7A* | CTT CAC CAG GCA GGA CAA | GGT TCT TCA GGT CGT AGT TGT G |
| *KLF1* | CGT ATG GCT TCT CCC CTG | CAG AGG ATC CAG GTG TGA TAG |
| *GATA1* | CAG GAC AGG CCA CTA CCT AT | CTG ACA ATC AGG CGC TTC TT |
| *GATA2* | CAA GGC TCG TTC CTG TTC A | GCC CAT TCA TCT TGT GGT AGA |
| *MYB* | CCA ATT ATC TCC CGA ATC GAA C | ACA AGC TCT ATC ACT CTC TGA TC |
| *ATF4* | CCT CCA ACA ACA GCA AGG A | GCA TGG TTT CCA GGT CAT CTA |
| *HBA* | TGG ACC CGG TCA ACT TCA AG | TCA CAG AAG CCA GGA ACT TGT |
| *HBB* | GAA GGC TCA TGG CAA GAA AG | CAC TGG TGG GGT GAA TTC TT |
| *HBG* | TCA CAG AGG AGG ACA AGG CTA | GCT TTA TGG CAT CTC CCA AG |
