# Supplementary material for: Induction of fetal hemoglobin: Lentiviral shRNA knockdown of HBS1L in β0-thalassemia/HbE erythroid cells
Source: PLoS One. 2023 Mar 8;18(3):e0281059. doi: 10.1371/journal.pone.0281059 (PMC9994754; doi:10.1371/journal.pone.0281059)
Supplement: Attachment — Submitted filename: Respoonse to Reviewers.docx [file pone.0281059.s005.docx]

5. Review Comments to the Author

Please use the space provided to explain your answers to the questions above. You may also include additional comments for the author, including concerns about dual publication, research ethics, or publication ethics. (Please upload your review as an attachment if it exceeds 20,000 characters)

Reviewer #1: In this paper, Peerapittayamongkol and colleagues build upon prior studies where they found correlation between a SNP in HBS1L and level of fetal hemoglobin (HbF) as well as disease severity in patients diagnosed with HbE β-thalassemia. As expression of HBS1L was reported to be reduced in culture differentiated erythroblasts from individuals with hereditary persistence of fetal hemoglobin (HPFH), the authors hypothesized that shRNA-mediated knockdown of HBS1L may lead to increased production of HbF in culture-differentiated erythroblasts of heathy volunteers and HbE β-thalassemia patients. That said, studies conducted on erythroblasts from HPFH individuals did not account for variations in genetic architecture of the globin locus or differential effects of known HbF repressor proteins. Also to the contrary, Sankaran and colleagues reported that complete loss of all major isoforms of HBS1L had no impact on hematological parameters or HbF levels (Sankaran et al, Blood (2013) 122 (23): 3845–384).

Here the authors, found that expression of HBS1L in culture-differentiated erythroblasts of heathy volunteers and HbE β-thalassemia patients was essentially indifferent, however, this experiment was only designed to assess total HBS1L rather than discriminate between total and alternative splice variants of this gene product (V1, V2, V3). This is particularly important as the authors provide an argument for significance of the V3 isoform in the discussion as a contradiction to prior published findings (Sankaran et al, Blood (2013) 122 (23): 3845–384). They went on to show that shRNA knockdown of HBS1L increased levels of gamma-globin mRNA and HbF in differentiated erythroblasts of heathy volunteers and HbE β-thalassemia patients without significant impact on erythroid differentiation or cell morphology. That said, they did not evaluate the expression of fetal hemoglobin per cell by flow cytometry, ability of these cultured cells to reach full maturity as demonstrated by enucleation, or compare total hemoglobin for HBS1L KD β-thalassemia cells versus healthy donors. Finally, the authors do not expand upon these initial finds by delving into the potential responsible mechanism(s) to include changes in expression of: 1) known HbF repressors or activators, 2) chromatin modifiers, or 3) erythroid-specific transcription factors that may be affected by loss of a protein involved in mRNA stability, which limits the overall significance of these findings.

Major comments

lines 65 – 68: The description of HBS1L function is difficult to appreciate from the provided text. Please revise these two sentences to clarify this information.

We have rephrased “HBS1L functions in combination with PELOTA in identifying the stalled ribosome complexes on truncated mRNAs and causing them to be separated into subunits. [15]. Additionally, this incident starts mRNA degradation pathways. “

Supplementary Table 1. It is preferable to use “healthy donor” as an alternative to “normal”. Patients in this study are also “normal” they just have thalassemia. Please replace N = normal with HD = Healthy donor throughout the manuscript to include figures and figure legends.

We have replaced "healthy donors" with "normal" in S1 Table and everywhere else.

lines 116 – 141. The description of the shRNAs, construction of lentiviral vector, and production and transduction can be combined into a single section.

In the section entitled “ShRNA-carrying lentivirus vector development, selection, and transduction’ contained the explanation of the shRNAs (A list of target sequences shown in Table 1), the development of the lentiviral vector, and production and transduction.

lines 148 – 162. The description of RNA isolation, reverse transcription and qPCR can be combined into a single section.

We combined them into the section entitled “RNA isolation, reverse transcription, and quantitative polymerase chain reaction (qPCR)”.

Figures 1 and 2. Please combine these data into a single figure where panel A demonstrates results for the 3 healthy donors and panel B for the 4 thalassemia patients. It would be beneficial if the scale of the Y-axis were the same for panels A and B so that differences in mRNA expression can be easily appreciated by the reader. This figure also needs a descriptive legend summarizing the information presented in each panel. Please replace N = normal with HD = Healthy donor for the reason described above. Finally, it would be beneficial to place into context the mean expression level ± S.D. for the healthy donors vs. patients at each time point. Were any of the differences in expression statistically significant?

We had trouble adjusting the scale of the previous Figures 1 and 2. To merge them, we created one graph, Fig 2, utilizing different lines and symbols representing HD and patient groups. There were none that attained statistical significance at any time. The expression level was presented as mean± S.D.

Figures 3 and 4. Please combine these data into a single figure where panel A demonstrates results for HBS1L mRNA level and panel B protein level. This figure also needs a descriptive legend summarizing the information presented in each panel and could benefit from a schematic diagram shown the timing of lentiviral transduction, cell expansion and differentiation as well as collection of samples for isolation of mRNA and protein. It is odd that levels of HBS1L mRNA were unchanged relative to untreated cells by qPCR but protein levels appear to be reduced in both the cytoplasmic and nuclear fractions even though equivalent amounts of protein appear to be loaded as demonstrated by lamin and actin. Do the authors have any explanation for this result?

Figures 3 and 4 were now Figs 3A and 3B. The description of this Figure was added. This could be due to variable off-target response, where we observed a 26% reduction in the transcript levels in certain individuals. This may correspond to the reduction in the protein levels of 16%.

The timing of lentiviral transduction, cell growth and differentiation, as well as the collection of samples for mRNA and protein isolation, were schematically shown in Fig 1.

Transduction with lentiviral shRNA was able to induce a nonspecific response due to the perturbation of host miRNAs (doi: 10.1128/JVI.00503-18), where we observed a 26% reduction in the transcript levels in certain individuals. This might correspond to the reduction in the protein levels of 16% in the Western blot.

Figures 5 and 6. Please combine these data into a single figure where panel A - D demonstrates flow cytometry results and panels E and F photomicrographs of cell cytospins. This figure also needs a descriptive legend summarizing the information presented in each panel.

We cooperated with the request, and Fig 4 provided an illustration of them.

Figures 7, 8, and 9. Please combine these data into a single figure where panel A and B demonstrates results for alpha, beta and gamma globin expression for the healthy donors and thalassemia patients, respectively. It would be beneficial if the scale of the Y-axis were the same for panels A and B so that differences in expression can be easily appreciated by the reader. Figure 8 can be presented as panel C with results plotted as bars demonstrating mean ± SD for each condition as an alternative to the line graph which is somewhat confusing. Finally, HPLC results can be presented as panels D and E, respectively. This figure also needs a descriptive legend summarizing the information presented in each panel.

We cooperated with the request, and Fig 5 provided an illustration of them.

Minor comments

line 29: remove space in “Hb E” to read “HbE” and remove the word “erythroid”

line 30: replace “on” with “of”

line 39: Change “Asea” to “Asia”

line 45: remove space in “Hb E” to read “HbE”

line 47: citation (6) appears to have a reduced font size

line 47: remove space in “Hb F” to read “HbF”

line 49: replace “transplantation” with “transplant”

line 54: replace “amending” with “amend”

lines 45-57: combine text into a single paragraph

line 59: remove the word “in” and replace “reveals” with “revealed”

line 61: remove space in “Hb E” to read “HbE”

line 62: remove “the” and remove space in “Hb F” to read “HbF”

line 64: remove “the” and remove space in “Hb F” to read “HbF”

line 70: remove space in “Hb E” to read “HbE”

line 75: remove space in “Hb F” to read “HbF”

line 84: remove the word “normal”

line 85: remove space in “Hb E” to read “HbE”

line 86: replace “normal” with “volunteers” or “subjects”

line 90: “Mmononuclear” should read “Mononuclear”

line 121: % appears to have a reduced font size

line 282: replace “expressions” with “expression”

lines 283 and 286: replace “normal” with “healthy controls” or “healthy subjects”

line 302: replace “normal” with “healthy”

We have corrected all misspellings.

Reviewer #2: Hemoglobin disorders including beta-thalassemia can be cured by hematopoietic stem cell (HSC) gene therapy with beta-globin gene addition or short hairpin RNA (shRNA)-based fetal hemoglobin (HbF) induction. BCL11A gene, LRF gene, HBS1L-MYB region, and other HPFH (hereditary persistence of fetal hemoglobin) mutations are main targets for HbF induction. In this study, the authors demonstrated that shRNA-based knockdown of the HBS1L gene can induce HbF expression in CD34+ cell-derived erythroid cells in vitro (from healthy donors and beta0-thalassemia / hemoglobin E patients); however, the HbF induction levels were relatively low at 5-10% in healthy donor cells (1% at the baseline) and 35-40% in patients’ cells (20% at the baseline).

Major points

1. The authors should demonstrate the shRNA-target sites (shRNA1 and shRNA2) on a schema of the HBS1L gene and RNA variants. In addition, the authors should demonstrate the target sequences of shRNA1 and shRNA2.

In S1 Fig, a schema displaying the shRNA-target locations on HBS1L variants was shown.

Table1 provided a list of the shRNA1, shRNA2, and shNTC target sequences.

2. The authors should compare HbF amounts at the protein level in CD34+ cell-derived erythroid cells between HBS1L knockdown and BCL11A knockdown.

We are unable to solve this problem. One of our colleagues used the same methodology as the current investigation to carry out a *BCL11A* knockdown (unpublished data). However, none of the transduced cells survived.

In HD erythroblasts, *HBS1L* knockdown caused an increase in %HbF but no change in BCL11A expression.

3. The authors should evaluate erythroid-specific transcriptional factors (such as GATA1, GATA2, KLF1, BCL11A, and MYB) in erythroid cells with or without knockdown of HBS1L.

In Fig. 6, the effects of HBS1L knockdown on the expression of transcription factors related to erythroid lineage, including ATF4, BCL11A, GATA1, GATA2, KLF1, and MYB, were depicted.

4. The authors should demonstrate a schema of methods regarding cell type, conditions of lentiviral transduction, and methods of erythroid differentiation for Figures 1-2, Figures 3-4, Figures 5-6, and Figures 7-9.

The schema of methods was now depicted in Fig 1

5. In Figure 1-2, the authors should demonstrate whether erythroid cells are sufficiently matured for hemoglobin analysis, such as cell surface analysis for CD71 and CD235a.

According to the plan shown in Fig. 1, hemoglobin measurement was carried out on day 14 and cell surface analysis on day 12. The results in Fig. 4 could be used to infer the cell maturity for hemoglobin analysis.

6. In Figure 3, the authors should demonstrate HBS1L RNA amounts in un-transduced cells.

The expression of HBS1L in untransduced cells was set to one, as demonstrated in Equation 2.

7. In Figure 4, the authors should explain why HBS1L protein amounts were reduced in the shNTC control.

Transduction with lentiviral shRNA was able to induce a nonspecific response due to the perturbation of host miRNAs (doi: 10.1128/JVI.00503-18), where we observed a 26% reduction in the transcript levels in certain individuals. This might correspond to the reduction in the protein levels of 16% in the Western blot.

8. In Figures 3-4, Figures 5-6, and Figures 7-9, the authors should demonstrate transduction efficiency (such as vector copy number) in transduced cells.

Due to the scarcity of DNA samples, we were unable to calculate the vector copy number. However, puromycin was present in the culture media at a dosage of 1 µg/mL, rendering all untransduced cells incapable of surviving.

Alternately, we reported the amount of HBS1L transcript that remained after knockdown in Fig5A and 5B.

9. In Figure 5, the author should demonstrate vector copy number (or HbF induction) among each fraction, if lentiviral transduction efficiency in bulk cells is not ~100%.

The answer was similar to that of question 8.

10. In Figure 6, the authors should measure the percentage of enucleation among groups.

The percentage of enucleated cells among groups was presented in Fig. 4G. (previous Figure 6 is now a part of Fig.4)

11. In Figure 7, the authors should demonstrate gamma-globin RNA amounts in un-transduced cells.

The expression of gamma globin mRNA in untransduced cells was set to one. The globin expression of transduced cells was presented as relative to that of untransduced cells Fig.5A and 5B.

12. In Figure 8, the authors should demonstrate a graph of the average and standard deviation with statistical analysis regarding the absolute amounts (percentages per total hemoglobin) of HbF, HbA, and HbA2/E.

Fig. 5C showed a graph of the average and standard deviation together with statistical analysis of the percentages of HbF, HbA, and HbA2/E.

Minor pints

1. In Methods, the authors should define the pLL3.7 lentiviral vector.

We have modified “the pLL3.7 lentiviral vector into "the pLL-Puro, a modified version of the pLL3.7 lentiviral plasmid. In this plasmid, the EGFP gene was replaced by a puromycin-resistant gene. [18]. "

2. In Methods, the authors should explain the more detailed methods for HPLC.

We added “This cation exchange HPLC technique uses an increasing sodium phosphate gradient buffer to separate different hemoglobin variants. Hemoglobin was measured using absorbance at 415 nm, and correction for turbidity was done by the absorbance at 690 nm [20]. Each 6.5-minute assay detects the most prevalent aberrant hemoglobin variations while also providing quantitative findings for the percentages of HbA2/E and HbF.”

3. The authors should combine Figures 1 and 2, Figures 3 and 4, Figures 5 and 6, as well as Figures 7, 8, and 9.

The previous Figure 1 and 2 were combined into Fig 2, Figure 3 and 4 into Fig 3, Figure 5 and 6 into Fig 4 and Figure 7, 8 and 9 into Fig 5, respectively.

4. In the Figure 3 legend, the author should explain how to calculate HBS1L RNA amounts.

The present version of manuscript was in Fig 3. *HBS1L* expression of transduced cells was presented as relative to that of untransduced cells (Equation 2). In other words, the expression of *HBS1L* in untransduced cells was set to one.

6. PLOS authors have the option to publish the peer review history of their article (what does this mean?). If published, this will include your full peer review and any attached files.

If you choose “no”, your identity will remain anonymous but your review may still be made public.

Do you want your identity to be public for this peer review? For information about this choice, including consent withdrawal, please see our Privacy Policy.

Reviewer #1: No

Reviewer #2: No

[NOTE: If reviewer comments were submitted as an attachment file, they will be attached to this email and accessible via the submission site. Please log into your account, locate the manuscript record, and check for the action link "View Attachments". If this link does not appear, there are no attachment files.]

While revising your submission, please upload your figure files to the Preflight Analysis and Conversion Engine (PACE) digital diagnostic tool, https://pacev2.apexcovantage.com/. PACE helps ensure that figures meet PLOS requirements. To use PACE, you must first register as a user. Registration is free. Then, login and navigate to the UPLOAD tab, where you will find detailed instructions on how to use the tool. If you encounter any issues or have any questions when using PACE, please email PLOS at figures@plos.org. Please note that Supporting Information files do not need this step.

In compliance with data protection regulations, you may request that we remove your personal registration details at any time. (Remove my information/details). Please contact the publication office if you have any questions.
