# Supplementary material for: Induction of fetal hemoglobin: Lentiviral shRNA knockdown of HBS1L in β0-thalassemia/HbE erythroid cells
Source: PLoS One. 2023 Mar 8;18(3):e0281059. doi: 10.1371/journal.pone.0281059 (PMC9994754; doi:10.1371/journal.pone.0281059)
Supplement: Attachment — Submitted filename: Response to Reviewers.docx [file pone.0281059.s006.docx]

PONE-D-21-35741R1

Induction of fetal hemoglobin: Lentiviral shRNA knockdown of HBS1L in β0-thalassemia/HbE erythroid cells.

PLOS ONE

Dear Editor,

I want to thank Reviewers 1 and 2 for addressing the important issues in the earlier submission. I sincerely appreciate Reviewer 3's remarks on our reviewer response and the interest to discuss about the relationship between GATA-1 and the *HBS1l-MYB* intergenic region. Additional discussion has been added to line number 375-390 of the present revision.

ChiP experiment suggests that GATA-1 interacts with the core intergenic *HBS1L-MYB* region, regulating the expression of MYB in a way that is selective in erythroid lineage. The shRNA method, in contrast to CRISPR-Cas9, acts at the transcriptional level without affecting the genomic DNA. As we demonstrated, *GATA-1*'s transcripts were essentially identical, therefore it should continue to be expressed. HbF levels rise despite CRISPR-Cas9-mediated *ZNF410* reduction without changing *GATA-*1 levels (Mol Cell, 2021. 81(2): p. 239-254.e8). It's possible that GATA-1 and the *HBS1L-MYB* intergenic region had nothing to do with the rise in HbF after *HBS1L* knockdown.

I am incredibly appreciative of PLOS ONE's article submission procedure.

Best regards,

Chayanon Peerapittayamongkol, PhD, MD

Department of Biochemistry, Faculty of Medicine Siriraj Hospital,

Mahidol University, Thailand
